# Persister Cells Form in the Plant Pathogen *Xanthomonas citri* subsp. *citri* under Different Stress Conditions

**DOI:** 10.3390/microorganisms9020384

**Published:** 2021-02-14

**Authors:** Paula M. M. Martins, Thomas K. Wood, Alessandra A. de Souza

**Affiliations:** 1 Department of Chemical Engineering, Pennsylvania State University, University Park, PA 16802, USA; pmmm@outlook.com.br; 2 Biotechnology Laboratory, Centro de Citricultura Sylvio Moreira, Instituto Agronômico de Campinas, Rodovia Anhanguera Km 158, Cordeirópolis-SP 13490-000, Brazil

**Keywords:** persistence, citrus canker, phytopathogen, citrus

## Abstract

Citrus canker disease, caused by the bacterium *Xanthomonas citri* subsp. *citri* is a constant threat to citrus-producing areas. Since it has no cure, agricultural practices to restrain its dissemination are essential to reduce the economic damage. Hence, increased knowledge of the basic aspects of *X. citri* biology could lead to more efficient management practices that can eliminate dormant bacteria in the field. The dormant cells, also referred to as persisters, are phenotypic variants with lowered metabolism, which in turn leads to tolerance to antimicrobials and undermines existing control approaches. We show here that *X. citri* forms persisters, identifying triggers for this phenotype, including antibiotics, high temperature, and metals (copper and zinc), which increase persistence rates by 10–100 times. The antioxidant N-acetylcysteine reduced copper and zinc-induced persisters, but not those induced by tetracycline, indicating that oxidative stress may be an important inducer of *X. citri* persistence. In addition, we found that metabolism-independent drugs like cisplatin and mitomycin C are able to eliminate *X. citri* persistent cells, as well as copper, at high concentrations. Specific amino acids like proline and isoleucine interfered with the physiological balance of the dormancy in *X. citri*, stimulating or preventing persister resuscitation. Taken together, we discover chemicals that can induce, wake, and kill *X. citri* persister cells; these results provide insights that should be considered for more efficient integrated control management in the field.

## 1. Introduction

Citrus canker, caused by the bacterium *Xanthomonas citri* subsp. *citri* is one of the most devastating diseases found in citrus-growing areas [1,2]. Despite successful eradication programs in Florida (USA) [3] and Sao Paulo (Brazil) [2], these two major orange producers in the world face constant citrus canker epidemics [4,5]. For different reasons, both eradication programs were halted in Brazil and Florida [6]; hence, the citrus canker has increased substantially in these areas, and now growers are expected to live with this disease endemically [2]. There is no cure, and chemical control is limited to the application of cupric compounds [4,7], which also have limited effectiveness [8]. Therefore, strict management practices are essential to keep field levels of *X. citri* at low levels [2,5].

*X. citri* is a biotrophic phytopathogen [9] and, as such, relies entirely on its plant host to survive. It infects all citrus varieties, which have different levels of susceptibility [10]. *X. citri* has limited survival outside of the citrus plant, does not utilize insect vectors or an alternate host, so dissemination is via wind-blown water droplets, which carry the bacterium that oozes from canker lesions. In addition, human and tool movements between infected and healthy orchards are important mechanisms for bacteria spreading [1,3]. Despite its relatively simple life cycle, citrus canker outbreaks are recurrent, meaning current efforts to suppress dissemination are not very effective. Therefore, it is imperative to better understand *X. citri* survival after the use of cupric bactericides to design more sustainable management practices to decrease the environmental and economic damage caused by the disease in the field.

*X. citri*, like most bacteria, survive most adverse conditions. Nutrient deprivation, heat and cold stresses, as well as a myriad of dangerous chemicals, like antibiotics, are examples of challenges microorganisms face continuously. Hence, strains are selected to withstand these adverse conditions, primarily through genetic mutation and/or the introduction of foreign genetic material (like plasmids), which allow cells to survive under otherwise prohibitive conditions. Once acquired, the resistance genes are stably passed to offspring, giving rise to resistant strains. However, another widespread survival mechanism is the persistence phenotype.

Characterized by a general suppression of bacterial metabolism, this dormancy phenomenon was first described in the 1940s [11,12], when a small subpopulation of *Staphylococcus aureus* cells entered into a dormant state, protecting them from the antibiotic. Later, reports presented a so-called “viable, but non-culturable” state (“VBNC”) in *Escherichia coli* and *Vibrio cholerae* [13], which was further described in many other bacterial species. Some inconsistencies in the nomenclature of these “dormant” phenotypes have occurred in the following years. Recent works have clarified the nature of persistence, showing that, at least for *E. coli*, the viable cells in the VBNC state are persister cells [14] and that persistence stems from ribosome dimerization [15]. In addition, advances in the resuscitation of persisters using single-cell microscopy have shown that they wake in a heterogeneous manner when exposed to carbon sources (rather than spontaneously) based on their ribosome content [16] and that specific chemicals can wake/resuscitate different bacterial strains [17,18]. Notably, when persisters resuscitate, they grow at the same growth rate as the wild-type strain [14].

The basic principle behind persistence is that many antimicrobials rely on active metabolic pathways [19]; consequently, by a general metabolic arrest, these chemicals do not find their targets and do not harm the dormant cells. The occurrence of persistence seems to be ubiquitous among bacteria, under both nutrient and antibiotic stress conditions. Even archaea become persisters [20].

The persistence has been thoroughly analyzed among medically-relevant bacteria, but its occurrence in phytopathogens is poorly understood, with only a few bacterial species studied to date. For example, copper or chlorine exposure results in unculturable cells for some phytopathogens such as *Erwinia amylovora* [21,22,23], *Xylella fastidiosa* [24,25] and *Xanthomonas campestris* [26]. The occurrence of these unculturable cells (so-called VBNCs) in phytopathogens was first described in *Ralstonia solanacearum* [27], and the cause of these phenotypic variants is low temperatures [28] and starvation [29]. Curiously, oxidative stress is pivotal to the unculturability for both *Erwinia amylovora* [30] and *Ralstonia solanacearum* [31] since catalase supplementation changes the culturability profiles.

Copper and zinc are potent inducers of oxidative stress in living cells [32,33,34,35,36]; hence, they are likely linked to persistence and unculturable cells in agriculture. These metals can induce oxidative stress by the production of hydroxyl radicals by the Haber-Weis and Fenton reactions [37,38]. This imbalanced oxidative environment can irreversibly damage lipids, nucleic acids and proteins, which can be fatal to live cells [37,38]. Metals cannot be synthesized or degraded, and their concentration needs to be strictly balanced: its excess can prevent bacterial growth, and complex metalloregulatory pathways have evolved [39].

The current knowledge on persistence in phytopathogens has been reviewed [40], highlighting that, despite persistence occurring in the field, agricultural management practices largely neglect persister cells. This can result in the inadvertent production of these cells, which are more tolerant to regular control measures. In addition, since persister cells may not be killed by the application of many chemicals, special approaches are needed for their eradication. Here we show that *X. citri* cells become persistent in specific situations, and we suggest management practices that may lower the impact of their occurrence in the field.

## 2. Materials and Methods

### 2.1. Bacterial Strains and Growth Conditions

All experiments were performed using the sequenced strain *Xanthomonas citri* subsp. *citri* 306 [41]. All incubations were done at 30 °C and 250 rpm using rich Nutrient Both Yeast medium-NBY (0.5% peptone, 0.3% meat extract, 0.2% yeast extract, 0.2% K2HPO4, and 0.05% KH_2_PO_4_), unless otherwise specified. For solid media, agar (1.2%) was added to NBY, and incubations were carried out at 30 °C for at least 48 h. Antibiotics and other chemicals were supplemented when necessary, using 1× MIC (minimal inhibitory concentration). The MICs were obtained using NBY rich medium inoculated with 10 µL of overnight *X. citri* cultures (turbidity of ~1.0 at 600 nm), plus each of the chemicals, tetracycline (1 µg/mL); ciprofloxacin (0.5 µg/mL); copper (160 µg/mL); zinc (322 µg/mL); cisplatin (50 µg/mL); mitomycin C (0.25 µg/mL) and NAC (8 mg/mL). A negative control (only NBY, without the addition of any chemical) was also included. The test tubes were incubated overnight (30 °C, 200 rpm), and the MIC was determined as the lowest concentration that prevented bacterial growth by visual inspection.

### 2.2. Persistence Induced by Chemicals and Persister Cells Killing

Twenty-five milliliters of overnight grown *X. citri* cells (turbidity of 0.8 to 1.0 at 600 nm) were pretreated with copper (1 × MIC), zinc (1 × MIC) or tetracycline (10 × MIC) for varying periods (from 15–60 min) to induce persister cells. Afterward, cells were harvested, and the pellet suspended in a fresh NBY medium supplemented with 50 µg/mL ciprofloxacin (100 × MIC) and incubated for an additional 3 h to kill non-persister cells. Subsequently, aliquots were taken for serial dilution and CFU counting. Control cultures (25 mL) were directly treated with ciprofloxacin, without any pretreatment.

For the “killing” experiments, ciprofloxacin was replaced by cisplatin, mitomycin C or copper, and incubation times varied for each experiment.

### 2.3. Persistence Induced by Temperature

After reaching turbidity ~0.8–1.0, 25 mL of *X. citri* culture was transferred to 37 °C, 250 rpm shaker for 1, 3 and 6 h. Thereafter, cells were harvested, and the pellet was suspended in fresh NBY medium supplemented with 50 µg/mL ciprofloxacin (100 × MIC) and incubated for 3 h in order to kill non-persister cells. Control cultures were directly treated with ciprofloxacin, without heat-treatment.

### 2.4. Persister Resuscitation by Amino Acids

*X. citri* cells were grown to a turbidity ~0.8–1.0, and persisters were obtained by a 45 min pretreatment with tetracycline (10 × MIC), followed by 3 h treatment with ciprofloxacin (100 × MIC), as previously described. Cultures were serially diluted 10,000-fold (10^−4^) and 100 µL was plated on M9 solid medium supplemented with one of the following amino acids groups: #1 (Ala, Arg, Cys, Phe, Ser), #2 (Gly, His, Thr, Val, Tyr), #3 (Asn, Ile, Lys, Pro, Trp) or #4 (Asp, Glu, Gln, Leu, Met). Both M9 broth and the amino acids we used were as previously reported [42]. Plates were observed daily until day 4 when colonies could be first observed. The plates where colonies first appeared was considered as an indication of which amino acids combination were more efficient in resuscitating persister cells. To identify which specific amino acids was the most important in resuscitating persister cells, the same experiment was repeated, under the same conditions, but now using agar plates containing individual amino acids from group 3 (Asn, Ile, Lys, Pro, Trp) and 4 (Asp, Glu, Gln, Leu, Met) [42] that presented faster-growing colonies.

### 2.5. Antioxidant Effect of N-Acetylcysteine (NAC) on Metal-Induced Persisters

Cells were grown overnight in NBY medium until turbidity of ~0.8 to 1.0, and 25 mL was centrifuged; the pellet resuspended in 25 mL fresh NBY medium supplemented with NAC (1 × MIC) and copper (1 × MIC) and incubated for 30 min. Cells were harvested, resuspended in fresh NBY supplemented with ciprofloxacin (100 × MIC) and incubated for 6 h. Afterward, aliquots were taken for serial dilution and CFU counting. Control cultures followed the same procedure but were pretreated only with copper (1 × MIC, positive control) or without pretreatment (negative control).

### 2.6. Statistical Analysis and Data Presentation

Experiments were conducted with at least two biological replicates. For each time- point assessed, we calculated a median value, which we used to calculate the standard deviation. Data presented as “fold increase in survival” were obtained by dividing the CFU number obtained by a given treatment by the CFU number of the respective control. For statistical significance analysis, we used a *t*-test versus the control conditions and considered a minimum of 0.05 for the *p*-values. To better represent the statistical significance of the data shown as “fold increase in survival,” a *t*-test was conducted using directly the CFU numbers obtained during the experiment. For clarity, we presented graphic results for persisters (subjected to “pretreatments” with inducers) in relation to control (without the “pretreatment” with inducer). These results are shown in *Y*-axis as “fold increase in survival”, being the fold increase always in comparison to the control that had no persister-induction.

## 3. Results

### 3.1. X. citri Triggers Persister Cell Formation under Antibiotic Stress

In order to study persistence in bacteria, it is necessary to increase its proportion in a given population. As a proof of concept, we applied the same method previously utilized in *E. coli* [18,43,44]. The basic principle behind this technique is that if persister cells are formed by given stress, they can withstand a subsequent treatment with another antibiotic and are killed to a lesser extent than control cells (those not receiving the persister-inducing stress).

**Figure 1:**
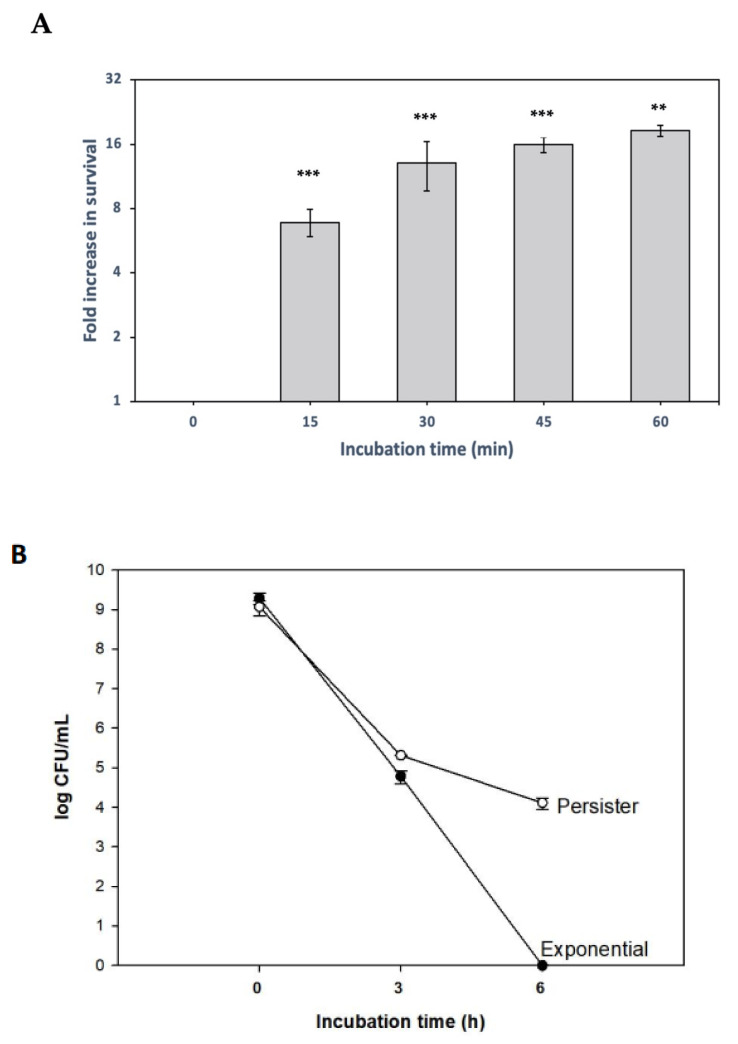
Persister cell induction by inhibiting translation and biphasic killing curve. (**A**) Exponentially growing *X. citri* cultures pretreated with the translation inhibitor tetracycline for 15, 30, 45 and 60 min, followed by ciprofloxacin treatment (50 µg/mL, 3 h, to kill non-persisters). Samples were serially diluted for colony-forming unit (CFU) counting to determine fold changes (relative to control without tetracycline). Results are shown as the mean of biological replicates. The symbols * (*p* < 0.05) and *** (*p* < 0.001) indicate significant differences using Student’s *t*-test. (**B**) Time course of cell death during ciprofloxacin treatment (50 µg/mL) in persisters (after tetracycline pretreatment) and exponential cells (without tetracycline pretreatment).

To initiate the persister study with *X. citri*, we used the antibiotic tetracycline, which blocks translation, and therefore induces a persistence state by blocking protein synthesis [43]. Then, a ciprofloxacin treatment was followed to kill non-persister cells. Therefore, *X. citri* cultures were grown in NBY media, and when they reached the mid-exponential phase, they were exposed to 10 × MIC tetracycline (10 µg/mL), for 15 to 60 min, centrifuged, and the pellet was resuspended in NBY supplemented with 100 × MIC ciprofloxacin (50 µg/mL). Indeed, there was an increase in cell survival among those cultures that were previously exposed to tetracycline before the ciprofloxacin treatment (Figure 1A). After only 15 min of tetracycline pretreatment, the amount of surviving cells was almost 10-fold higher than the control culture (only ciprofloxacin treatment, without tetracycline), after 3 h ciprofloxacin challenge, and from 30 to 60 min, the survivor ratio was slightly higher (Figure 1A).

We sought to determine how these persister cells would behave after prolonged ciprofloxacin treatment. The experiment was performed as before (Figure 1A), but at this time, a 45 min tetracycline incubation was used. Afterward, the ciprofloxacin challenge was carried out for 6 h, and the colony-forming unit (CFUs) was obtained for both the tetracycline pretreated and the control cultures (Figure 1B). After 6 h of ciprofloxacin treatment, no survivors were recovered in the control culture, while the tetracycline pretreated culture presented 10^4^ CFU/mL. A characteristic biphasic killing curve was seen, an expected result since persisters are more tolerant to antibiotic treatments. Therefore, we conclude that the inhibition of protein translation can induce persister cell formation in the plant–pathogen *X. citri*.

### 3.2. Field Stressors Such as Copper, Zinc and High-Temperature also Induce Persisters Cells in X. citri

**Figure 2:**
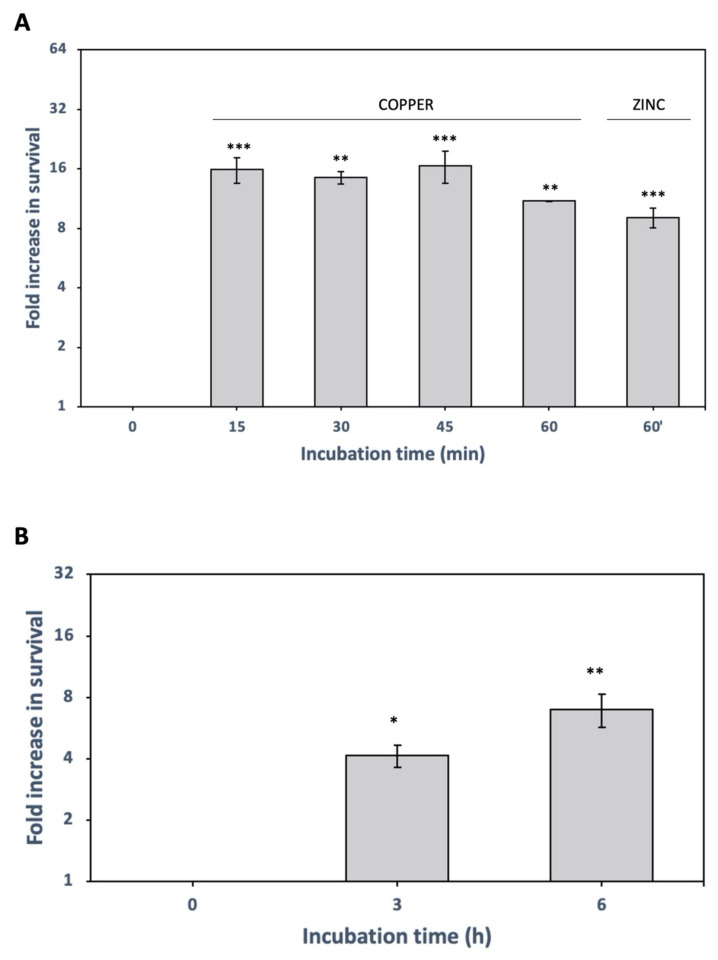
Induction of *X. citri* persisters after pretreatment with copper, zinc, and high temperature. (**A**) *X. citri* cultures pre-treated with copper 1 × minimal inhibitory concentration (MIC) (160 µg/mL) for 15, 30, 45 and 60 min or with zinc 1 × MIC (322 µg/mL) for 60 min, before ciprofloxacin treatment (50 µg/mL, 3 h). Fold changes in CFU in relation to control (without copper or zinc pretreatment) are shown as the mean of biological replicates and the symbols *** (*p* < 0.001), ** (*p* < 0.01) indicate significant differences using Student’s *t*-test. (**B**) Cultures were grown at 30 °C, and after reaching the mid-log phase, flasks were transferred to 37 °C for 3 h and 6 h before ciprofloxacin treatment (50 µg/mL, 3 h, to kill non-persisters). Fold changes in CFU counting are shown (relative to control without heat-treatment). Results are shown as the mean of biological replicates and symbol * (*p* < 0.05) and ** (*p* < 0.01) indicate significant differences using Student’s *t*-test.

To investigate if *X. citri* persister cells are induced by stressors they face in the field, we tested copper, which is widely used for *X. citri* control [2], zinc, which was recently proposed to control *X. citri* [45], and high temperature [46]. When we attempted to induce persistence using 10 × MIC tetracycline after 10 × MIC copper (1600 µg/mL) or 5 × MIC copper pretreatment, no cells survived due to the high copper concentrations. Persistence was only achieved for *X. citri* when 1 × MIC (160 µg/mL) copper pretreatment was used before 10 × MIC tetracycline. Hence, we tested ciprofloxacin (50 μg/mL, 3 h) treatment after a 1 × MIC of copper pretreatment at different incubation times (Figure 2A) to verify the development of persister cells. Among all copper pretreated cultures, we observed more than 10 times more survivors after 3 h ciprofloxacin killing treatment when compared to the control (no copper pretreatment). This ratio was stably maintained regardless of how long the copper pretreatment (15 to 60 min) (Figure 2A). In addition, we also found that a 60 min pretreatment with zinc at 1 × MIC (322 µg/mL) induces *X. citri* persistence under the same treatment conditions (Figure 2A). Since our goal was only to determine if zinc could induce persister cell formation like copper, we selected only one time-point to address this question. We observed that zinc induced persistence, increasing cell survival 10 times after ciprofloxacin treatment (Figure 2A). Therefore, we found that, besides their unequivocal antibacterial activity [45,47], metals can induce *X. citri* persistence.

Citrus is planted in tropical and subtropical regions where temperatures near 37 °C are common, and *X. citri* must withstand such environments. Thus, we hypothesized that the induction of persistence by high temperature would be one mechanism *X. citri* might use to remain viable under high-temperature field conditions. To test this hypothesis, *X. citri* cells were cultured at 30 °C to turbidity at 600 nm ~0.8–1.0 and were exposed to 37 °C from 1 to 6 h before ciprofloxacin treatment. After 3 h of ciprofloxacin treatment, we observed that cells pre-exposed to the higher temperature were more tolerant to the antibiotic since we obtained around 4 to 7 times more survivors than the control, depending on how long the heat-treatment was applied (Figure 2B). We conclude that besides chemical stress (tetracycline, copper, zinc), physical stress (high temperature) can also induce the formation of persister cells in *X. citri*.

### 3.3. X. citri Persister Cells Are Killed by Cisplatin, Mitomycin C, and High Copper Concentrations

Even with the suppression of metabolic routes (usually targeted by conventional antimicrobial molecules), persister cells can be killed by chemicals that do not interfere directly with metabolism and whose actions are independent of growth. At least two compounds are reported to kill persister cells: cisplatin [48] and mitomycin C [49,50]. In addition, since the copper killing mechanism for microorganisms is possibly independent of growth [51], we investigated the behavior of *X. citri* persister cells when exposed to these three chemicals: cisplatin, mitomycin C, and copper.

**Figure 3:**
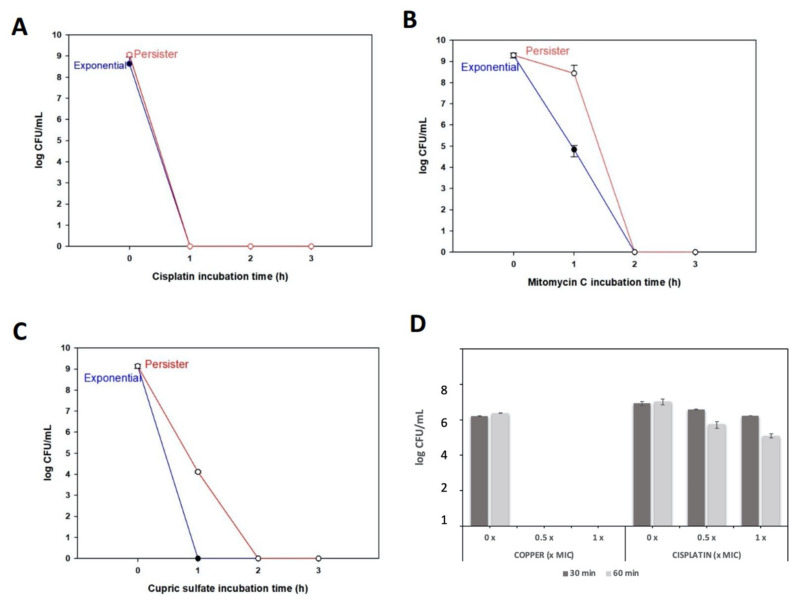
Persister cells killing by chemicals. For panels A–C, chemicals were used at 5 × MIC concentration in NBY medium. (**A**) Cisplatin (250 µg/mL); (**B**) mitomycin C (2.5 µg/mL) and (**C**) copper (800 µg/mL). (**D**) To compare the efficiency of copper versus cisplatin to kill persisters, bacterial suspensions of persisters were treated with 0, 0.5 or 1 × MIC of cisplatin and copper (1 × MIC copper = 160 µg/mL; 1 × MIC cisplatin = 50 µg/mL). After 30 min and 60 min incubations, samples were taken for CFU counting.

*X. citri* persister cells were obtained using tetracycline as the inducer (45 min, as shown in Figure 1B), and after this, the cultures were submitted to treatments with the different molecules, using a 5 × MIC concentration. Cisplatin killed persisters and exponentially growing cells at very similar rates, without any colonies observed after 1 h (Figure 3A). Mitomycin C also killed exponentially growing and persister cells, but complete eradication was observed only after 2 h (Figure 3B). In contrast, copper took longer to exterminate persister cells compared to exponentially growing cells, since persisters cells were killed after 2 h and exponential cells after 1 h of (Figure 3C). Noteworthy, for all cases, persisters were undetectable within 2 h (Figure 3A–C). This is a very different scenario than observed for ciprofloxacin treatment, where it was necessary to use a much higher concentration (~100 × MIC) and even after 6 h, persister cells were still detectable, although exponential cells were no longer present (Figure 1B). Therefore, cisplatin, mitomycin C, and copper are effective for killing *X. citri* persister cells.

To compare the killing potential of copper as a persister killing agent without the interference of growth media, we obtained persister cells by pretreating *X. citri* with tetracycline (Figure 1A), but, this time, the cells were washed and suspended in 0.85% NaCl buffer. To this bacterial suspension of persisters, copper was added at 32 µg/mL and 160 µg/mL (which corresponds to 0.2 × MIC and 1 × MIC, respectively). For comparison, the same experiment was performed with the addition of cisplatin instead of copper. We observed that after only 30 min, the lowest copper concentration (0.5 × MIC) eradicated *X. citri* persisters, while for cisplatin treatments, even after 60 min of incubation and using a higher concentration (1 × MIC), cisplatin could not eliminate these cells (Figure 3D). Overall, these results show that copper, depending on the environmental condition, can act as a persister inducer but can also kill persisters. Differences in killing by copper using rich media versus using NaCl buffer are probably due to copper associating with the abundant organic material present in rich media, which may reduce the amount of free copper that can effectively act on cells.

### 3.4. Cu and Zn Ions Induce Persistence by Oxidative Stress

**Figure 4:**
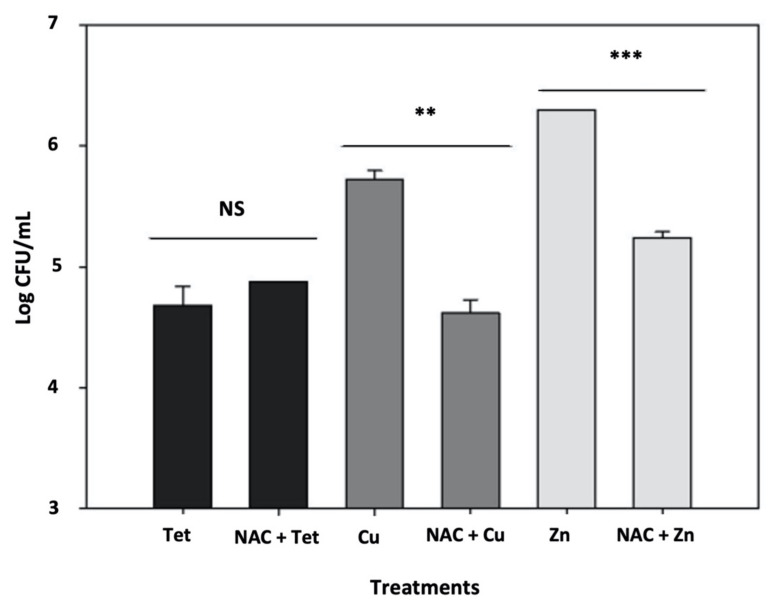
N-acetylcysteine (NAC) prevents metal-induced persister formation. Tetracycline-induced rates of persister cells (“Tet” black bar) are not affected by the addition of NAC during the pretreatment (“NAC + Tet”, black bar). Copper and zinc-induced rates of persistence (respectively “Cu”, dark gray bar; and “Zn” light gray bar) are affected by concomitant addition of the antioxidant NAC (“Cu + NAC”, dark gray bar; and “Zn + NAC” light gray bar). Results are shown as the mean of biological replicates and the symbols ** (*p* < 0.01) and *** (*p* < 0.001) indicate significant differences using Student’s *t*-test. NS indicates the absence of significative difference.

Since copper induces oxidative stress and since we found oxidative stress induces persistence by 12,000-fold in *E. coli* [52], we hypothesized that these metals could trigger persistence through oxidative stress. Therefore, if metals induce persistence through oxidative stress, an antioxidant may prevent or lower the persistence levels. As N-acetylcysteine (NAC) is a potent antioxidant [53] and is also used to control *Xanthomonas* spp. [54,55], we used NAC to investigate whether it can reduce the metal-induced persistence in *X. citri*. Indeed, we observed that when copper or zinc are present together with NAC, a significant decrease in the persistence rate is observed (Figure 4). As expected, this effect was not observed when tetracycline was used to induce *X. citri* persisters, indicating that: (1) this antibiotic does not induce persistence through oxidative stress and (2) metallic compounds may induce persistence through oxidative stress.

### 3.5. Specific Amino Acids Affect X. citri Persister Cell Resuscitation

**Figure 5:**
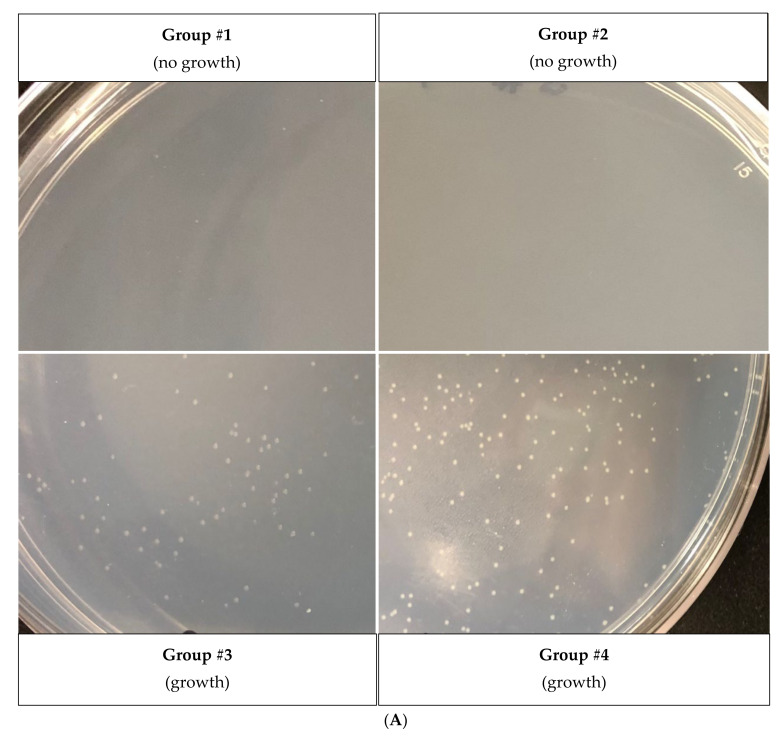

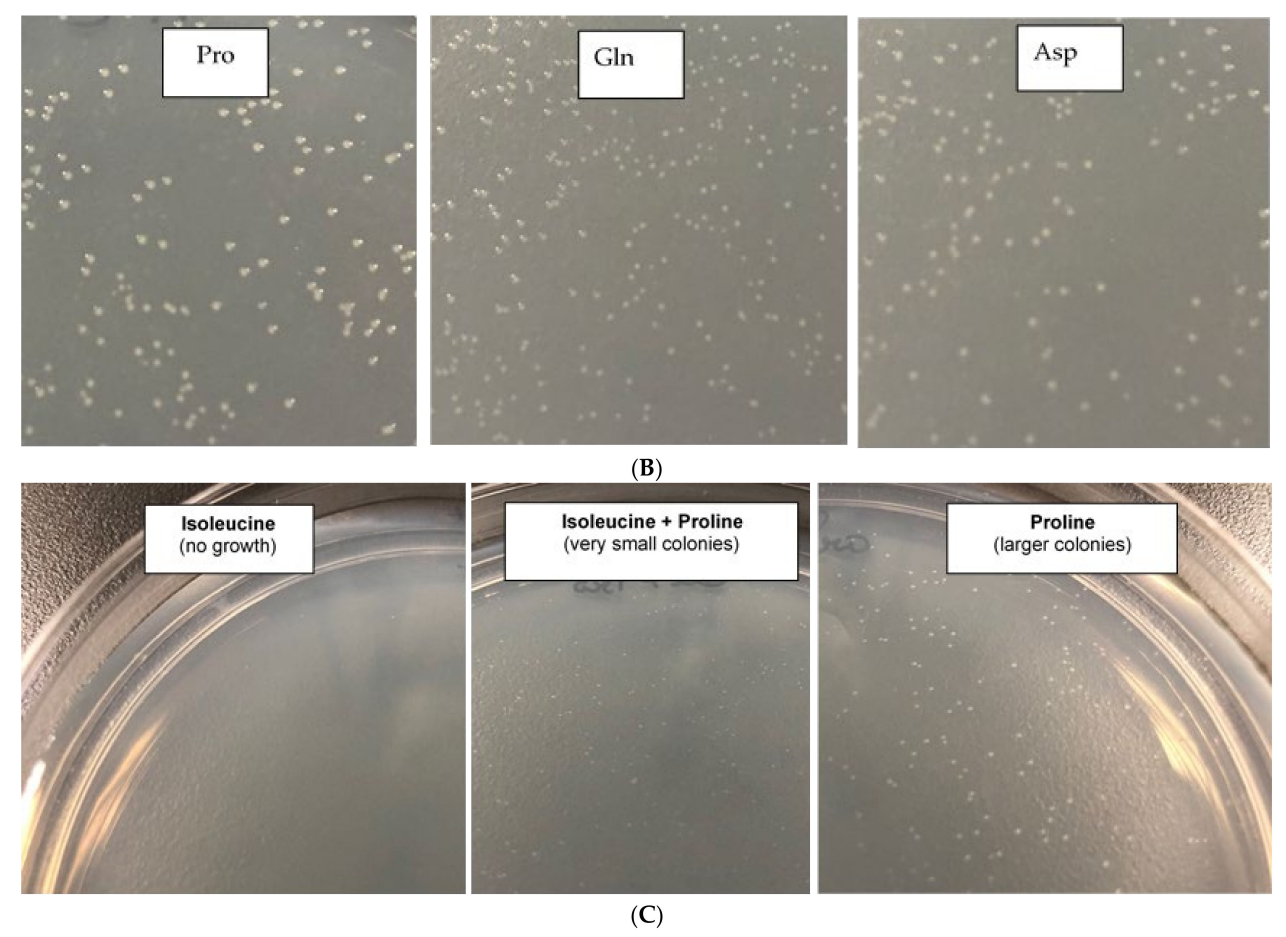
Effect of each group of amino acids on the regrowth of *X. citri* persister cells (**A**) amino acids group #1 (Ala, Arg, Cys, Phe, Ser); group #2 (Gly, His, Thr, Val, Tyr); group #3 (Asn, Ile, Lys, Pro, Trp) and group #4 (Asp, Glu, Gln, Leu, Met). (**B**) Same experiment as shown in A, but with M9 minimal medium containing individual amino acids of the fast-growing group #3 and group #4. Photographs show colonies observed with proline (group #3), glutamine (group #4) and aspartic acid (group #4). (**C**) To observe the inhibitory effect of isoleucine on *X. citri* persisters, a suspension of *X. citri* persister cells (same experiment as in A) were plated on M9-agar plates supplemented with only isoleucine; isoleucine + proline or only proline.

As a strategy to survive adverse conditions, the entry into persistence must be accompanied by a complimentary, sensory-based mechanism that will allow the cell to resume growth after stress, a method that has been established for *E. coli* [18]. Therefore, we tested whether different amino acids could also differentially influence *X. citri* resuscitation. For this purpose, persister cells were induced by tetracycline (followed by a ciprofloxacin treatment to kill non-persisters) and plated on an M9 solid medium supplemented with one of the following amino acid groups: #1 (Ala, Arg, Cys, Phe, Ser), #2 (Gly, His, Thr, Val, Tyr), #3 (Asn, Ile, Lys, Pro, Trp) or #4 (Asp, Glu, Gln, Leu, Met) [42]. We observed that group #4 sustained faster and better growth of persisters since more and larger colonies were present in these plates, followed by group #3 of amino acids (Figure 5A).

The next step was the screening of individual amino acids of group #4 to identify which one was responsible for inducing *X. citri* resuscitation. We decided to also evaluate the amino acids of group #3, which was the second-best group to resuscitate the persisters. Curiously, when individually screening the 10 different amino acids of groups #3 and #4, we found that the best amino acid to wake *X. citri* persister cells, i.e., the one that led to larger colonies, was proline, which was among group #3, and not #4 (Figure 5B). Among group #4, the best amino acids were Gln and Asp (Figure 5B), but proline from group #3 was even better. In addition, among all these individual amino acids screened, isoleucine plates (of group #3 of amino acids) were the only ones that did not have any *X. citri* colonies. We, therefore, hypothesized that maybe, isoleucine could have partially inhibited the waking of *X. citri* persisters in group #3 (Figure 5A), masking proline activity. In order to test this hypothesis, persister cells were obtained as described before, and the bacterial suspension was plated on different combinations of proline and isoleucine. Indeed, plates containing only proline yielded larger colonies (Figure 5C) than plates containing proline and isoleucine combined, which had much smaller colonies. Again, plates containing only isoleucine had no colonies.

## 4. Discussion

Citrus canker may seem, at first glance, a “simple” disease. Unable to survive outside of plants, it is not clear how bacterial population and disease rates rapidly increased after many years of suppression, nor is it clear how these inocula could have survived in the field enduring harsh environmental conditions. Based on the results presented in this study, we suggest that persistence in *X. citri* might be an essential biological step that has been preventing conventional management from successfully eradicating canker disease.

In the present work, we studied the persister phenotype in *X. citri*, one of the most important phytopathogenic bacteria for the citrus industry. We used standardized methodology, largely used for *E. coli* persistence studies [49,51], to determining specific triggers that can: (1) induce persistence (chemicals, high temperature), (2) kill persisters (copper, mitomycin C, cisplatin), (3) induce resuscitation/waking of persister cells (proline), and (4) prevent persisters from waking (isoleucine).

We found that copper, the main chemical used for plant disease control in the field [56] can both induce and kill persister cells in *X. citri* depending on the conditions used. The recommended concentrations of metallic copper to control citrus canker in the field is 0.5–0.7 g/L [57], therefore copper is used in the field at higher concentrations than we used in the present work. However, it is virtually impossible to precisely determine the copper concentration *X. citri* cells will be exposed to in the field. After being deposited on plant surfaces, copper products may be washed away from the leaves by rain and other meteorological events and may be deposited in water bodies and soils where other toxic effects can be observed [58]. In addition, copper can easily bind to organic compounds, changing its availability to bacterial cells on the leaves [59,60,61]. Therefore, residual amounts of copper could lead to dormancy, inducing persistence cycles in *X. citri* and requiring even higher amounts of chemicals to eradicate these still viable cells from the field.

Contrary to the widespread notion that persisters cannot be killed [62], we demonstrate that other chemicals besides copper can also eradicate *X. citri* persister cells. As already demonstrated for *E. coli* persisters, we found that molecules with a metabolism-independent mode of action such as cisplatin [48] and mitomycin C [49] can be used to control *X. citri* persister. However, due to their cost and toxicity, these compounds are not practical for large-scale use to control phytopathogens in the field.

The potential physical stresses that can induce persistence in bacteria are frequently overlooked. Since citrus orchards are grown under environments of high temperature, and *X. citri* cannot grow and complete its cell cycle above 35–37 °C [46], heat stress is one of the main challenges *X. citri* cells must overcome. This finding points to an interesting scenario, where daily heat-cold cycles may be regulating cell physiology and influencing dynamic disease processes. Apparently, *X. citri* cells may enter into persistence during the warmest periods of the day, when both stomata closure and plant defenses would impede successful infection [63]. Therefore, a subpopulation of cells that are exposed to these harsh environmental conditions can enter into the persister state when the energetic cost to successfully infect the host would be too high. Furthermore, if cells enter into the persistence state, they become more tolerant to antimicrobial compounds applied to citrus orchards, raising the question of whether these applications should be shifted to colder periods of the day.

We also found that the small antioxidant molecule NAC can prevent metal-induced persistence, shedding light on how we can more readily control persister cells in the field. NAC is a sustainable alternative to control *Xanthomonas* spp. Diseases that affect both citrus (citrus canker) and tomato (bacterial spot of tomato) [54,55]. Since metallic compounds can create an imbalanced internal oxidative environment [51], we presume that NAC could be acting directly as an antioxidant molecule, lowering oxidative stress [53] and blocking persister induction, which can be triggered by oxidative stress [30,40,64]. This “anti-persistence” effect has been previously suggested for *Mycobacterium* spp. [65,66] and our data support this previous finding. Of note, the combination of NAC and copper has better results in the control of both citrus canker and bacterial spot disease of tomato [54,55].

Finally, besides chemical inducers and killers of persistent cells, we also looked for molecules that could specifically wake *X. citri* persisters. Based on the finding that alanine is a signal recognized by *E. coli* persisters to restart growth [18], we identified proline as a signal to wake *X. citri* persisters. Curiously, proline is also the best amino acid to wake *Pseudomonas aeruginosa* from persistence [17]. Moreover, we observed that isoleucine has the opposite effect, blocking persister resuscitation, even when proline is also present, which is, to the best of our knowledge, a first report that an amino acid can prevent persister waking. It is interesting to note that valine inhibits *E. coli* growth due to the internal accumulation of an isoleucine precursor [67], which may involve RelA/ppGpp pathways [68]. Nevertheless, the reasons why these amino acids present these opposing roles are still unknown and require further investigation.

Overall, this work presents a basis for future studies on *X. citri* persistence to identify chemical and additional physical elements that can induce, kill, prevent and modulate persister waking. We recently found that the alarmone ppGpp directly causes the formation of *E. coli* persisters by dimerizing ribosomes [15] and that persister cells resuscitate by sensing nutrients through chemotaxis/sugar transporters, reducing cAMP, and stimulating ribosome activity [16,18], so perhaps these pathways hold as well for *X. citri*. This study opens new insights to better understand basic aspects of the biology of a previously ignored stage of this phytopathogen’s life cycle. Indeed, in-planta experimentation is still needed to confirm the extent to which persistence impacts disease control in the field, but this is beyond the scope of our study. Nevertheless, we expect that the knowledge of persister cell behavior under chemical and physical stresses can be used for further improvement of plant disease management in the field in order to obtain a more efficient and sustainable disease control.

## Data Availability

Data is contained within the article or supplementary material.

## References

[1] Gottwald T.R., Graham J.H., Schubert T.S. (2002). Citrus Canker: The Pathogen and Its Impact. Plant Health Prog..

[2] Behlau F., Fonseca A.E., Belasque J. (2016). A comprehensive analysis of the Asiatic citrus canker eradication programme in São Paulo state, Brazil, from 1999 to 2009. Plant Pathol..

[3] Graham J.H., Gottwald T.R., Cubero J., Achor D.S. (2004). *Xanthomonas axonopodis* pv. *citri*: Factors affecting successful eradication of citrus canker. Mol. Plant Pathol..

[4] Gochez A.M., Behlau F., Singh R., Ong K., Whilby L., Jones J.B. (2020). Panorama of citrus canker in the United States. Trop. Plant Pathol..

[5] Behlau F. (2020). An overview of citrus canker in Brazil. Trop. Plant Pathol..

[6] Gottwald T.R., Irey M. (2007). Post-hurricane Analysis of Citrus Canker II: Predictive Model Estimation of Disease Spread and Area Potentially Impacted by Various Eradication Protocols Following Catastrophic Weather Events. Plant Health Prog..

[7] Gottwald T.R., Timmer L. (1995). The efficacy of windbreaks in reducing the spread of citrus canker caused by *Xanthomonas campestris* pv. *citri*. Trop. Agric..

[8] Behlau F., Belasque J., Graham J.H., Leite R.P. (2010). Effect of frequency of copper applications on control of citrus canker and the yield of young bearing sweet orange trees. Crop Prot..

[9] Garavaglia B.S., Thomas L., Zimaro T., Gottig N., Daurelio L.D., Ndimba B., Orellano E.G., Ottado J., Gehring C. (2010). A plant natriuretic peptide-like molecule of the pathogen *Xanthomonas axonopodis* pv. *citri* causes rapid changes in the proteome of its citrus host. BMC Plant Biol..

[10] De Carvalho S.A., de Carvalho-Nunes W.M., Belasque J., Machado M.A., Croce-Filho J., Bock C.H., Abdo Z. (2015). Comparison of Resistance to Asiatic Citrus Canker Among Different Genotypes of Citrus in a Long-Term Canker-Resistance Field Screening Experiment in Brazil. Plant Dis..

[11] Hobby G.L., Meyer K., Chaffee E. (1942). Observations on the Mechanism of Action of Penicillin. Exp. Biol. Med..

[12] Bigger J.W. (1944). Treatment of staphylococcal infections with penicillin by intermittent sterilisation. Lancet.

[13] Xu H.-S., Roberts N., Singleton F.L., Attwell R.W., Grimes D.J., Colwell R.R. (1982). Survival and viability of nonculturable *Escherichia coli* andVibrio cholerae in the estuarine and marine environment. Microb. Ecol..

[14] Kim J.-S., Chowdhury N., Yamasaki R., Wood T.K. (2018). Viable but non-culturable and persistence describe the same bacterial stress state. Environ. Microbiol..

[15] Song S., Wood T.K. (2020). ppGpp ribosome dimerization model for bacterial persister formation and resuscitation. Biochem. Biophys. Res. Commun..

[16] Kim J.S., Yamasaki R., Song S., Zhang W., Wood T.K. (2018). Single cell observations show persister cells wake based on ribosome content. Environ. Microbiol..

[17] Zhang W., Yamasaki R., Song S., Wood T.K. (2019). Interkingdom signal indole inhibits *Pseudomonas aeruginosa* persister cell waking. J. Appl. Microbiol..

[18] Yamasaki R., Song S., Benedik M.J., Wood T.K. (2020). Persister Cells Resuscitate Using Membrane Sensors that Activate Chemotaxis, Lower cAMP Levels, and Revive Ribosomes. iScience.

[19] Lewis K. (2010). Persister Cells. Annu. Rev. Microbiol..

[20] Megaw J., Gilmore B.F. (2017). Archaeal persisters: Persister cell formation as a stress response in *Haloferax volcanii*. Front. Microbiol..

[21] Ordax M., Marco-Noales E., López M.M., Biosca E.G. (2006). Survival strategy of *Erwinia amylovora* against copper: Induction of the viable-but-nonculturable state. Appl. Environ. Microbiol..

[22] Santander R.D., Català-Senent J.F., Marco-Noales E., Biosca E.G. (2012). In planta recovery of *Erwinia amylovora* viable but nonculturable cells. Trees.

[23] Ordax M., Biosca E.G., Wimalajeewa S.C., López M.M., Marco-Noales E. (2009). Survival of *Erwinia amylovora* in mature apple fruit calyces through the viable but nonculturable (VBNC) state. J. Appl. Microbiol..

[24] Rodrigues C.M., Takita M.A., Coletta-Filho H.D., Olivato J.C., Caserta R., Machado M.A., de Souza A.A. (2008). Copper resistance of biofilm cells of the plant pathogen *Xylella fastidiosa*. Appl. Microbiol. Biotechnol..

[25] Muranaka L.S., Takita M.A., Olivato J.C., Kishi L.T., de Souza A.A., Muranaka L.S., Takita M.A., Olivato J.C., Kishi L.T., Souza A.A. (2012). De Global Expression Profile of Biofilm Resistance to Antimicrobial Compounds in the Plant-Pathogenic Bacterium *Xylella fastidiosa* Reveals Evidence of Persister Cells. J. Bacteriol..

[26] Ghezzi S. (1999). Induction of the viable but non-culturable condition in *Xanthomonas campestris* pv. *campestris* in liquid microcosms and sterile soil. FEMS Microbiol. Ecol..

[27] Grey B.E., Steck T.R. (2001). The viable but nonculturable state of *Ralstonia solanacearum* may be involved in long-term survival and plant infection. Appl. Environ. Microbiol..

[28] Van Elsas J.D., Kastelein P., de Vries P.M., van Overbeek L.S. (2001). Effects of ecological factors on the survival and physiology of *Ralstonia solanacearum* bv. 2 in irrigation water. Can. J. Microbiol..

[29] Álvarez B., López M.M., Biosca E.G. Extended nutrient limitation influences *Ralstonia solanacearum* survival in natural water microcosms. Proceedings of the Current Research Topics in Applied Microbiology and Microbial Biotechnology.

[30] Santander R.D., Figàs-Segura À., Biosca E.G. (2017). *Erwinia amylovora* catalases KatA and KatG are virulence factors and delay the starvation-induced viable but non-culturable (VBNC) response. Mol. Plant Pathol..

[31] Kong H.G., Bae J.Y., Lee H.J., Joo H.J., Jung E.J., Chung E., Lee S.-W. (2014). Induction of the Viable but Nonculturable State of *Ralstonia solanacearum* by Low Temperature in the Soil Microcosm and Its Resuscitation by Catalase. PLoS ONE.

[32] Pandey N., Pathak G.C., Pandey D.K., Pandey R. (2009). Heavy metals, Co, Ni, Cu, Zn and Cd, produce oxidative damage and evoke differential antioxidant responses in spinach. Braz. J. Plant Physiol..

[33] Thounaojam T.C., Panda P., Mazumdar P., Kumar D., Sharma G.D., Sahoo L., Panda S.K. (2012). Excess copper induced oxidative stress and response of antioxidants in rice. Plant Physiol. Biochem..

[34] Hamed S.M., Zinta G., Klöck G., Asard H., Selim S., AbdElgawad H. (2017). Zinc-induced differential oxidative stress and antioxidant responses in *Chlorella sorokiniana* and *Scenedesmus acuminatus*. Ecotoxicol. Environ. Saf..

[35] Gardner S.P., Olson J.W. (2018). Interaction of copper toxicity and oxidative stress in *Campylobacter jejuni*. J. Bacteriol..

[36] Singh R., Cheng S., Singh S. (2020). Oxidative stress-mediated genotoxic effect of zinc oxide nanoparticles on *Deinococcus radiodurans*. 3 Biotech.

[37] Gaetke L.M., Chow C.K. (2003). Copper toxicity, oxidative stress, and antioxidant nutrients. Toxicology.

[38] Grass G., Rensing C., Solioz M. (2011). Metallic copper as an antimicrobial surface. Appl. Environ. Microbiol..

[39] Chandrangsu P., Rensing C., Helmann J.D. (2017). Metal homeostasis and resistance in bacteria. Nat. Rev. Microbiol..

[40] Martins P.M.M., Merfa M.V., Takita M.A., de Souza A.A. (2018). Persistence in phytopathogenic bacteria: Do we know enough?. Front. Microbiol..

[41] Da Silva A.C.R., Ferro J.A., Reinach F.C., Farah C.S., Furlan L.R., Quaggio R.B. (2002). Comparison of the genomes of two *Xanthomonas* pathogens with differing host specificities. Nature.

[42] Rodriguez R.L., Tait R.C. (1983). Recombinant DNA Techniques: An Introduction.

[43] Kwan B.W., Valenta J.A., Benedik M.J., Wood T.K. (2013). Arrested protein synthesis increases persister-like cell formation. Antimicrob. Agents Chemother..

[44] Martins P.M.M., Gong T., de Souza A.A., Wood T.K. (2020). Copper Kills *Escherichia coli* Persister Cells. Antibiotics.

[45] Graham J.H., Johnson E.G., Myers M.E., Young M., Rajasekaran P., Das S., Santra S. (2016). Potential of nano-formulated zinc oxide for control of citrus canker on grapefruit trees. Plant Dis..

[46] Sumares J.A.P., Morão L.G., Martins P.M.M., Martins D.A.B., Gomes E., Belasque J., Ferreira H. (2016). Temperature stress promotes cell division arrest in *Xanthomonas citri* subsp. *citri*. Microbiologyopen.

[47] Cabot C., Martos S., Llugany M., Gallego B., Tolrà R., Poschenrieder C. (2019). A Role for Zinc in Plant Defense Against Pathogens and Herbivores. Front. Plant Sci..

[48] Chowdhury N., Wood T.L., Martínez-Vázquez M., García-Contreras R., Wood T.K. (2016). DNA-crosslinker cisplatin eradicates bacterial persister cells. Biotechnol. Bioeng..

[49] Kwan B.W., Chowdhury N., Wood T.K. (2015). Combatting bacterial infections by killing persister cells with mitomycin C. Environ. Microbiol..

[50] Cruz-Muñiz M.Y., López-Jacome L.E., Hernández-Durán M., Franco-Cendejas R., Licona-Limón P., Ramos-Balderas J.L., Martinéz-Vázquez M., Belmont-Díaz J.A., Wood T.K., García-Contreras R. (2017). Repurposing the anticancer drug mitomycin C for the treatment of persistent *Acinetobacter baumannii* infections. Int. J. Antimicrob. Agents.

[51] Vincent M., Duval R.E., Hartemann P., Engels-Deutsch M. (2018). Contact killing and antimicrobial properties of copper. J. Appl. Microbiol..

[52] Hong S.H., Wang X., O’Connor H.F., Benedik M.J., Wood T.K. (2012). Bacterial persistence increases as environmental fitness decreases. Microb. Biotechnol..

[53] Aldini G., Altomare A., Baron G., Vistoli G., Carini M., Borsani L., Sergio F. (2018). N-Acetylcysteine as an antioxidant and disulphide breaking agent: The reasons why. Free Radic. Res..

[54] Qiao K., Liu Q., Xia Y., Zhang S. (2021). Evaluation of a Small-Molecule Compound, N-Acetylcysteine, for the Management of Bacterial Spot of Tomato Caused by Copper-Resistant *Xanthomonas perforans*. Plant Dis..

[55] Picchi S.C., Takita M.A., Coletta-Filho H.D., Machado M.A., de Souza A.A. (2016). *N*-acetylcysteine interferes with the biofilm formation, motility and epiphytic behaviour of *Xanthomonas citri* subsp.. citri. Plant Pathol..

[56] Lamichhane J.R., Osdaghi E., Behlau F., Köhl J., Jones J.B., Aubertot J.N. (2018). Thirteen decades of antimicrobial copper compounds applied in agriculture. A review. Agron. Sustain. Dev..

[57] Behlau F., Belasque J. (2014). Cancro Cítrico: A Doença e Seu Controle.

[58] Hippler F.W.R., Petená G., Boaretto R.M., Quaggio J.A., Azevedo R.A., Mattos D. (2018). Mechanisms of copper stress alleviation in Citrus trees after metal uptake by leaves or roots. Environ. Sci. Pollut. Res..

[59] Alva A.K., Graham J.H., Anderson C.A. (1995). Soil pH and Copper Effects on Young ‘Hamlin’ Orange Trees. Soil Sci. Soc. Am. J..

[60] Holm T.R. (1990). Copper complexation by natural organic matter in contaminated and uncontaminated ground water. Chem. Speciat. Bioavailab..

[61] Karlsson H.L., Cronholm P., Hedberg Y., Tornberg M., de Battice L., Svedhem S., Wallinder I.O. (2013). Cell membrane damage and protein interaction induced by copper containing nanoparticles-Importance of the metal release process. Toxicology.

[62] Kim J.S., Wood T.K. (2016). Persistent persister misperceptions. Front. Microbiol..

[63] Griebel T., Zeier J. (2008). Light regulation and daytime dependency of inducible plant defenses in *Arabidopsis*: Phytochrome signaling controls systemic acquired resistance rather than local defense. Plant Physiol..

[64] Santander R.D., Monte-Serrano M., Rodríguez-Herva J.J., López-Solanilla E., Rodríguez-Palenzuela P., Biosca E.G. (2014). Exploring new roles for the *rpoS* gene in the survival and virulence of the fire blight pathogen *Erwinia amylovora*. FEMS Microbiol. Ecol..

[65] Vilchèze C., Hartman T., Weinrick B., Jain P., Weisbrod T.R., Leung L.W., Freundlich J.S., Jacobs W.R. (2017). Enhanced respiration prevents drug tolerance and drug resistance in *Mycobacterium tuberculosis*. Proc. Natl. Acad. Sci. USA.

[66] Defraine V., Fauvart M., Michiels J. (2018). Fighting bacterial persistence: Current and emerging anti-persister strategies and therapeutics. Drug Resist. Updat..

[67] Yang C.R., Shapiro B.E., Hung S.P., Mjolsness E.D., Hatfield G.W. (2005). A mathematical model for the branched chain amino acid biosynthetic pathways of *Escherichia coli* K12. J. Biol. Chem..

[68] Varik V., Oliveira S.R.A., Hauryliuk V., Tenson T. (2016). Composition of the outgrowth medium modulates wake-up kinetics and ampicillin sensitivity of stringent and relaxed *Escherichia coli*. Sci. Rep..

